# Improved Adhesion Performance of Soy Protein-Based Adhesives with a Larch Tannin-Based Resin

**DOI:** 10.3390/polym9090408

**Published:** 2017-09-01

**Authors:** Mingsong Chen, Jing Luo, Ruiqing Shi, Jizhi Zhang, Qiang Gao, Jianzhang Li

**Affiliations:** 1 Key Laboratory of Wood Material Science and Utilization, Beijing Forestry University, Beijing 100083, China; chen_boss@bjfu.edu.cn (M.C.);luojing.rowe@gmail.com (J.L.); shiruiqing@bjfu.edu.cn (R.S.); 2 Ministry of Education, Beijing Key Laboratory of Wood Science and Engineering, College of Materials Science and Technology, Beijing Forestry University, Beijing 100083, China; 3 Key Laboratory for Liquid-Solid Structural Evolution and Processing of Materials (MOE), School of Materials Science and Engineering, Shandong University, 17923 Jingshi Road, Jinan 250061, China; zjzvip@sdu.edu.cn

**Keywords:** soy protein, larch tannin, crosslinking, interpenetrating network, water resistance, bonding strength, plywood

## Abstract

This study aimed to improve the bonding strength and water resistance of soy protein-based adhesives (SPAs) by modifying with larch tannin-based resins (TRs). This is especially important because of their eco-beneficial effects. The TR was characterized by Fourier Transform Infrared (FTIR) and Thermogravimetric/Derivative Thermogravimetric (TG/DTG) in order to demonstrate the formation of the self-crosslinking structure. Rheological properties, fracture morphology, solubility, and crosslinking density were characterized in detail. Three-ply poplar plywood was fabricated and the wet shear strength was measured. The experimental data showed that the addition of TR improved the moisture uptake, residual rate, and shear strength of SPA. This improvement was attributed to the crosslink reactions of TR with the relevant active functional groups of the side chains of soy protein molecules. The crosslinking structure joined with the TR self-crosslinking structure to form an interpenetrating network, which promoted a uniform and compact cured structure. The 5 wt % TR additions in the SPA was found to yield optimum results by improving the wet shear strength of the plywood by 105.4% to 1.13 MPa, which meets the interior-use plywood requirement. Therefore, the larch tannin could be applied in the modification of soy protein adhesive.

## 1. Introduction

Studies on eco-friendly materials that are based on agricultural biomass resources (e.g., proteins, polysaccharides, gelatin, lignin, and tannin) used for coatings, packaging, and furnishings have gained popularity. Thus, many manufacturers and researchers are eager to study novel environmentally-friendly materials in the wood industry [1,2]. Soy proteins have significant potential in the wood industry as a type of biodegradable, abundant, inexpensive, and environmentally-friendly biomass.

Soy protein-based adhesives (SPAs) are eco-friendly products and the resulting plywood, having very low emissions of formaldehyde, is able to easily meet the current requirements in terms of formaldehyde emissions [3,4]. But these adhesives have a low water resistance. Previous researchers have attempted to improve the performance of SPAs by denaturation [5] and crosslinking agents [6,7]. One of the most effective ways is by introducing crosslinking agents like synthetic latex [8], polyamidoamine epichlorohydrin resin (PAE) [9], and epoxide [10]. These crosslinking agents react with the –NH_2_, –OH, and other exposed active groups on the soy protein molecules to increase the crosslinking density of the cured adhesives, thus improving the performance of resultant adhesive. However, the use of these crosslinking agents have issues: (1) these crosslinkers are derived from fossil resources [11]; (2) the addition of these crosslinkers are large, normally the ratio of protein/crosslinker is 1.5:1 [12]; (3) the use of PAE and epoxide increase the brittleness of the resultant adhesive, which results in a low dry bond strength. Therefore, developing high-performance crosslinker from a biomass is important for reducing the reliance on fossil resource and the cost.

Tannin is one of the most abundant compounds extracted from biomass; it exists readily in nature, especially in the bark and fruit of many species of trees [13]. Tannins have become regularly used in the agricultural industry, the food industry, and the leather industry [14]. Tannins have a high reactivity that can react with aldehydes or epoxides to develop tannin-based resins (TRs) due to the existing natural phenolic compounds [15,16]. The uses of tannins that replace phenol to develop phenol formaldehyde resin have been extensively researched [17]. The high reactivity of the tannin is considered in the following study. Glyoxal was introduced to form a reaction with the tannins in order to avoid formaldehyde and decrease the reactivity. Resorcinol and glyoxal were combined with larch tannin to synthesize a tannin-based resin (TR), then the TR was characterized by FTIR and TG. TR was mixed with soy protein isolate (SPI) to develop a series of SPAs. The effects of the TR content on the properties of the adhesives and the plywood were investigated. These included the viscosity, the moisture uptake rate and the residual rate of the SPAs, functional groups, and the fracture surface micrographs of the cured adhesives. Three-ply poplar plywood specimens were fabricated with the adhesives and tested to determine the dry and wet shear strength. The experimental results aimed to propose a novel, effective approach for the modification of SPI in bioadhesives, as well as to provide complementary information about the preparation methods for future use.

## 2. Materials and Methods 

### 2.1. Materials

Soy protein isolate (SPI) with 3% moisture, 1% ash, and 96% protein were purchased from Yuwang Ecological Food Industry Co., Ltd. (Dezhou, China). Commercial larch tannin with 60.1% tannin content, 9.5% moisture, and 30.4% non-tannin, including polysaccharide, ash, were purchased from Tian’guan Biotech Co., (Nanyang, China). A poplar veneer with a moisture content of 8.0% and dimensions of 400 mm × 400 mm × 1.5 mm were purchased from a local plywood plant. Resorcinol, glyoxal (40 wt %), sodium hydroxide (NaOH), and the remaining chemicals were AR grade reagents that were purchased from Beijing Chemical Reagents Co., Ltd. (Beijing, China). 

### 2.2. Preparation of TR

TR was synthesized in accordance to Pizzi’s research [18]. The tannin (70 g) and deionized water (43.6 g) were placed in a three-necked flask equipped with a reflux condenser and a stirrer. The mixture was continuously stirred. Methanol (16.88 g) and resorcinol (29.66 g) were subsequently added at 25 °C, followed by the glyoxal solution (9.8 g, 40%) and the aqueous sodium hydroxide solution (8 g, 45%). The mixture was heated to 80 °C and maintained for 3 h, with a pH of 10.0 and then cooled to 25 °C. 

### 2.3. Preparation of SPAs

The SPI (12 g) in deionized water (88 g) was stirred for 20 min at 25 °C in order to form a homogeneous system. Different amounts (0, 2.5, 5.0, 7.5, and 10 g) of TR were mixed with the pure SPA. They were stirred for an additional 10 min at 25 °C to develop a series of adhesives. The resulting adhesives were labeled “SPA”, “SPA-TR2.5”, “SPA-TR5”, “SPA-TR7.5” and “SPA-TR10” in accordance to the different TR amounts.

### 2.4. Characterization of SPAs

#### 2.4.1. Rheology Properties

The viscosities of the pure SPA and the TR-modified SPA samples were measured by a HAAKE RotoVisco 1 rheometer (Thermo Scientific, Waltham, MA, USA) with a parallel plate fixture (PP35, 35 mm plate diameter). The distance and the shear rate were set to 1 mm and 10 s^−1^ for all measurements, respectively. All of the measurements were conducted in triplicate and then averaged.

#### 2.4.2. Fourier Transform Infrared (FTIR) Spectroscopy

The prepared TRs and adhesive samples were cured in an oven for several hours at 120 ± 2 °C until the weight remained constant. The larch tannin powder and the ground cured adhesive powder sample ground to 200 mesh were blended with KBr crystals with a mass ratio of 1/70. The sample folium was obtained using a micro tablet press. The FTIR spectra of the different samples were recorded using a Thermo Nicolet 6700 FTIR (Thermo Fisher Scientific, Waltham, MA, USA) from 400 to 4000 cm^−1^ with a 4 cm^−1^ resolution and 32 times scans.

#### 2.4.3. Scanning Electron Microscopy (SEM)

The different adhesive samples were cured completely in an oven at 120 ± 2 °C. The samples were then placed into a desiccator for two days before examination. The cured adhesives were artificially fractured and then the fractures were coated with gold. A Hitachi S-3400N (Hitachi Science Systems. Ltd., Ibaraki, Japan) scanning electron microscope was used to observe the fractured surfaces of the different adhesive samples.

#### 2.4.4. Moisture Uptake Measurement

The moisture uptake measurement of the cured adhesive was determined by gravimetric analysis. Six pieces of cured adhesives were placed in a constant temperature humidity chamber with 80% humidity and 50 °C. The weight of the cured adhesives (α) was measured every two hours until a constant weight (β) was obtained. The moisture uptake value was calculated by the following equation:

(1) $$\mathrm{Moisture}\;\mathrm{uptake}\;\mathrm{value}\;\left(\%\right)=\;\frac{Weight\;\left(\beta \right)-Weight\;\left(\alpha \right)}{Weight\;\left(\alpha \right)}\times 100\%$$

#### 2.4.5. Residual Rate Test

The adhesive samples were placed in an oven at 120 ± 2 °C until a constant weight (*M*) was obtained. The sample were then ground into 100 mesh powder (0.15 mm) by a ceramic mortar. To determine the residual rate, the cured adhesives were wrapped up in qualitative filter paper tightened with rubber bands and placed in glass beakers filled with distilled water. The samples were hydrolyzing for 6 h in an oven at 60 ± 2 °C dried at 105 ± 2 °C for 3 h, and then weighted (*m*). The residual rate was defined as *m* divided by *M*, see Equation (2). The average value of the residual rate was calculated from the six parallel samples:

(2) $$\mathrm{Residual}\;\mathrm{rate}\;\left(\%\right)=\;\frac{m\left(g\right)}{M\left(g\right)}\times 100\%$$

### 2.5. Preparation of the Plywood Samples

Three-ply poplar plywood samples were prepared by spreading 200 g·m^−2^ of glue on a single surface, where they were hot pressed at 120 °C with a hot press pressure of 1.0 MPa, for 315 s [19]. The plywood samples were then stored under ambient conditions (20 °C and 12% relative humidity) for a minimum of 24 h prior to testing.

### 2.6. Shear Strength Measurement of Plywood

The dry and wet shear strength was tested in accordance with the Chinese National Standard (GB/T 17657-2013) [20]. Twelve plywood specimens with the dimensions of 100 mm × 25 mm (bond zone of 25 mm × 25 mm) were steadily slotted from the two plywood panels. The specimens for wet shear strength measurement were soaked in water at 63 ± 2 °C for 3 h, and then dried at ambient temperature for 10 min before tension testing. The dry and wet shear strength were measured by a universal material testing machine with a speed of 10.0 mm/min. Wood failure was estimated in accordance with the standard method of determining the percentage of wood failure in adhesive-bonded joints. The reported shear strength was an average value of twelve specimens and the standard deviation was given. 

### 2.7. Statistic Analysis

The data in the current study were statistically evaluated using the statistical software package ANOVA, SPSS 24.0 (SPSS Inc., Chicago, IL, USA). The data are reported as the mean value ± standard deviation of the replicates. A single factor analysis of variance was conducted to differentiate significant differences among mean values of the data according to the least significant difference criteria (*p* < 0.05).

## 3. Results

### 3.1. Characteristic of Tannin and TR Samples

#### 3.1.1. Physical Properties of TR Samples

Physical properties of TRs mainly included the solid content, viscosity and pH value. As shown in Table 1, the solid content and viscosity of the TR were measured. The solid content of the resultant resin was 57.1% and the viscosity was 970 mPa·s at 25 °C. 

#### 3.1.2. FTIR Spectroscopic Analysis of Tannin and TR

**Figure 1:**
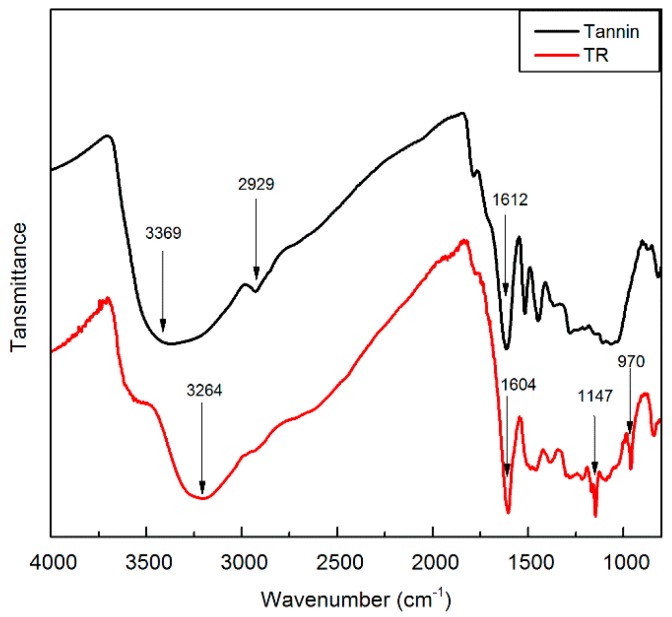
FTIR spectra of tannin/TR.

**Scheme 1:**
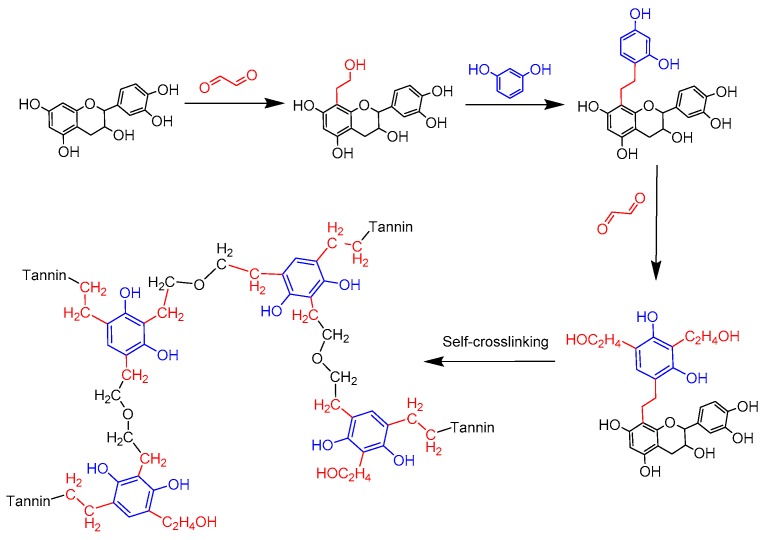
The reaction process of TR.

The FTIR spectra of the tannin and the TR are shown in Figure 1. There is a broad peak in the tannin spectra at the region of 3369 cm^−1^ that was the characteristic of hydroxyl groups that could contain water molecules; the small peak around 2929 cm^−1^ was due to aromatic C–H stretching vibrations; the absorption bands between 1620 cm^−1^ and 1450 cm^−1^ were caused by the deformation of aromatic C=C bonds in the aromatic rings [21]. The TR spectra showed a new absorption observed at 970 cm^−1^ that was attributed to the C–O stretching vibration in resorcinol. This indicated that the tannin reacted with resorcinol and glyoxal and formed a tannin-glyoxal-resorcinol oligomer [22]. The reaction process is shown in Scheme 1. The occurrence of substitution reaction between phenol and glyoxal and/or between phenol and resorcinol suggests that a reactive tannin-based bi-hydroxyethyl oligomer is formed. The formed oligomer contained large amounts of hydroxyethyl, which could react with itself to form a self-crosslinking structure and with the –NH_2_ on the protein to form a crosslinking structure. The other new peak present at 1147 cm^−1^ was the C–O–C stretching vibration, which indicated that these hydroxyethyl reacted with themselves by a polycondensation to formed ether bonds that connect the different tannin molecules. This was in accordance with Pizzi’s research [23].

#### 3.1.3. Thermal Behavior of Tannin and TR

**Figure 2:**
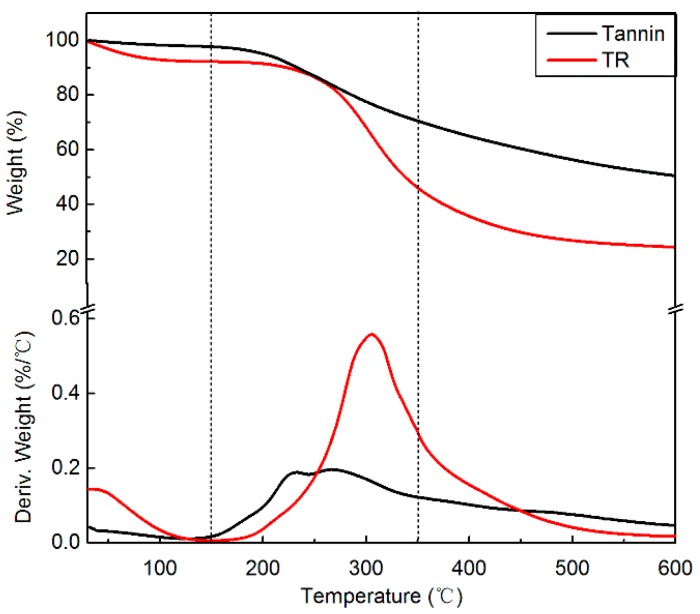
TG-DTG curves of tannin and TR.

The thermogravimetric (TG) and derivative thermogravimetric (DTG) curves of the tannin and the TR samples are shown in Figure 2. The tannin sample primarily decomposed within the range of 150–350 °C, which presented a significant mass loss. The tannin was a mixture of different compounds, which presented a wide range of degradation peaks in the curve of the tannin. There was an obvious peak around 300 °C that was observed in the curve of TR, which indicated that the TR had a different structure with a higher thermostability. This confirmed the result reported by Pizzi [24].

### 3.2. Viscosity of the Adhesive Samples

The viscosity of the different adhesives is shown in Table 2. While under the same shear rate, the viscosity of TR-SPA adhesive increased considerably with the TR addition increasing from 2.5 g to 10 g. The viscosity of the SPA was recorded at 111,900 mPa·s. The SPI molecule contained many hydrophilic groups, including –OH and –NH_2_. These groups interact in the water to form many intermolecular forces, which prevent movement between molecules. This results in a high viscosity. The viscosity of the SPA-TR2.5 adhesive decreased by 59.1% to 45,800 mPa·s after 2.5 g of TR was added. The molecular weight of the TR was much lower than that of protein. The TR molecule inset into the protein molecules and reduced their intermolecular forces, which reduced the viscosity. This observation was in agreement with the Yuan’s research [25]. The viscosity of the adhesive increased further as the additional TR increased. When 10 g of TR was added, the viscosity of the SPA-TR10 adhesive increased by 130.4% to 105,512 mPa·s compared with the SPA-TR2.5. This was because free sodium hydroxide in the TR unfolded the protein molecules under an alkaline condition (pH value < 10). This increased the intermolecular force and significantly increased the adhesive viscosity [26].

### 3.3. FTIR Spectroscopic Analysis of the Adhesvie Samples

**Figure 3:**
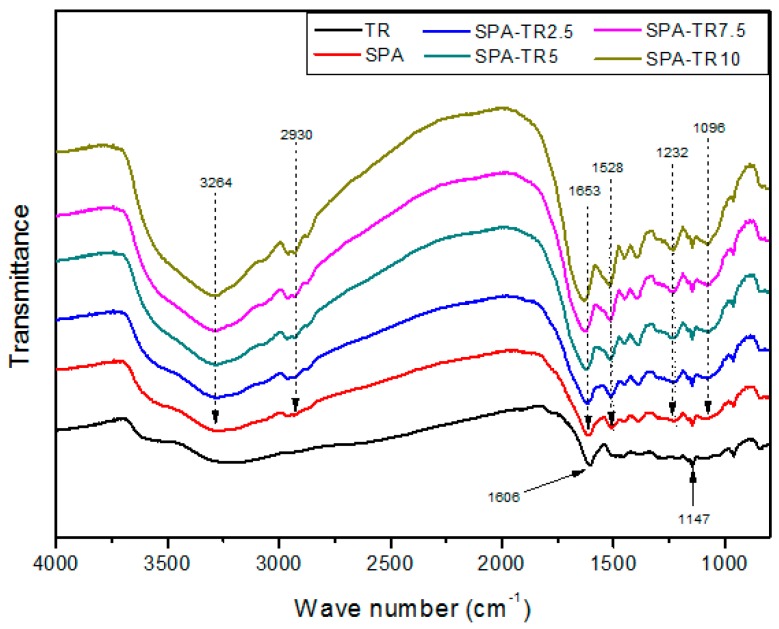
FTIR spectra of different adhesives.

The FTIR spectra of the TR, SPI, and the resulting SPAs are shown in Figure 3. The SPA spectra had a broad peak in the 3264 cm^−1^ region, which was the characteristic of the O–H and N–H groups. They were then able to form C–O and C–N bonds with the carbonyl group of the peptide linkage on the protein molecules. A peak at about 2930 cm^−^^1^ was attributed to the –CH_2_ group. The main absorption peaks at 1653, 1532, and 1234 cm^−1^ were usually characterized by the amide I (C=O stretching), amide II (N–H bending), and amide III (C–N and N–H stretching), respectively. This finding was in accordance with Zhao’s research [27]. The spectra of the SPAs with different TR additions had a peak that corresponded to the C–O bending at 1096 cm^−1^.

**Scheme 2:**
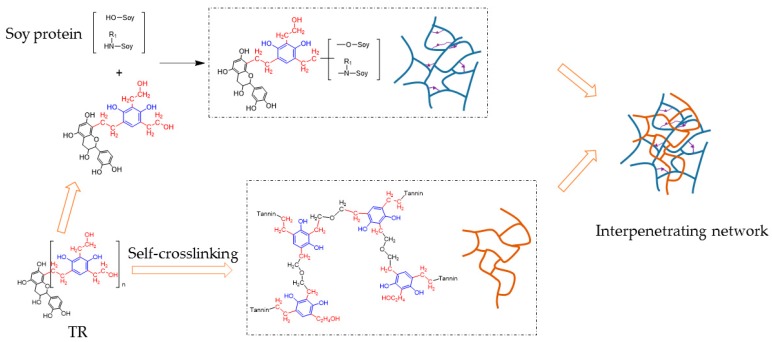
The reaction of TR/SPI.

The absorption peaks of amide III gradually increased with the raising of TR additions from 0 g to 5 g in the adhesives. This was attributed to the reaction between hydroxyethyl in TRs and –NH groups on the soy protein molecules, which formed the crosslinking network structure. In addition, peaks observed at about 1096 cm^−1^ gradually increased. This increase was attributed to (1) the reaction between the hydroxyethyl in the TRs and the hydroxyl on the soy protein molecules, which increased the crosslinking density of the resultant SPAs; (2) the formation of an interpenetrating network between the self-polycondensation network and the soy protein molecules network to further increase the crosslinking density, thus improving the water resistance of the resultant SPAs [28]. The reaction between TR and protein molecules is shown in Scheme 2. The occurrence of crosslinking reaction between amino groups on soy protein molecules and hydroxyethyl in TRs suggested that a crosslinking structure was formed. In addition, adjacent hydroxyethyls in TRs reacted to form an ether linkage, thus, forming another crosslinking structure. These two crosslinking structures were entangled mutually to form an interpenetrating network structure in hot-pressing process. The absorption peak of amide III became constant as the TR addition was increased from 5 g to 10 g, which indicated that the addition of TR was excessive. The excessive TR formed a low water resistance structure, which reduced the water resistance of the adhesive [29].

### 3.4. SEM Analysis of the Adhesive Samples

**Figure 4:**
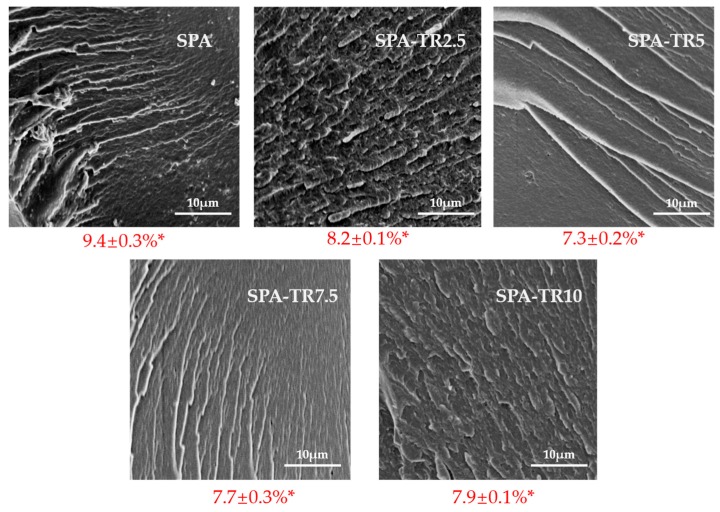
The fracture surface micrograph of different cured adhesives (* represents the moisture uptake of different cured adhesives).

The fracture surface micrographs of the cured adhesives are shown in Figure 4. A loose fracture surface with some cracks was seen in the SPA, which suggested that the inherent brittle and the cohesive failures were caused by the evaporation of a large quantity of water. These cracks would swell to break the bonds and resulted in the low water resistance of the adhesive [30]. No cracks were seen after 5 g of TR was added, and the fracture surface of the cured adhesive became smoother and more compact. This suggested that the TR and the soy protein formed a crosslinking network. These results confirmed the results discussed above (Figure 3). When the TR was increased to 10 g, the cracks and the disordered surface reappeared on the facture surface. This was due to the soy protein molecules decomposing as the TR addition increased. These decomposed protein molecules had many hydrophilic groups that filled in the TR and reduced the water resistance of the composite adhesive.

The moisture uptake of the different cured adhesive samples is also shown in Figure 4. The moisture uptake of the modified adhesives decreased, which suggested that the modified adhesives with TR addition were efficient in preventing moisture intrusion and improved moisture resistance. These findings are in agreement with Neto’s research [31]. As the TR addition went from 0 g to 5 g, the moisture uptake decreased from 9.4 to 7.3%. This indicated that the reduced surface roughness corresponded to the improved moisture resistance of the cured adhesive. As the TR addition went from 5 g to 10 g, the moisture increased from 7.3 to 7.9%. This indicated that the increased surface roughness corresponded to the deterioration of moisture resistance in the cured adhesive. The smooth surface of the adhesive SPA-TR5 was due to the increased adhesive fluidity and the fact that the TR modified the soy protein molecules, which formed a stable system.

### 3.5. Shear Strength of Plywood

The average residual rate of the cured adhesives and the shear strengths of plywood are shown in Table 3. The residual rate of the pure SPA was the lowest at 61.8%, which indicated that the pure SPA had the lowest crosslinking structure among the adhesives. Therefore, the wet shear strength of the plywood bonded with pure SPA was 0.56 MPa, which does not meet the interior-use plywood requirement. After introducing 5 g of TR, the residual rate of the SPA-TR5 adhesive was improved by 38.4% when compared to the pure SPA and the wet shear strength increased by 105.4% to a maximum value of 1.13 MPa. This significant increase was attributed to three reasons: (1) NaOH found in the TR increased the protein solubility and the content of ionic amino acids, which could improve the reactivity between protein molecules and crosslinkers [32]; (2) the formed self-crosslinking structure of the TR could form an interpenetrated network with cross-linked soy protein molecules, which would further increase the crosslinking density of the adhesive; (3) the C–O–C and C–N bonds that are formed by the reaction between the active groups on the soy protein molecules and the TR resulted in a solid structure that improves the water resistance of adhesive, which further increased the water resistance of the resultant plywood. The dry shear strength of the pure SPA was the lowest at 1.24 MPa. After introducing 5 g of TR, the dry shear strength increased by 83.1% to a maximum value of 2.27 MPa, which was attributed to the increased crosslinking density of SPAs and the formation of the glue nail structure in the wood interface because of the penetration of small molecules. After further introducing TR in SPAs, the dry shear strength decreased gradually, which was because the higher viscosity limited the uniformity of adhesive on the wood surface.

## 4. Conclusions

The TR effectively improved the water resistance of the SPA, as well as the wet shear strength of the resultant plywood. The following conclusions were drawn from the results obtained in this report:

(1) The addition of 5 wt % TR in SPA increased the residual rate of the resultant adhesive by 38.4% and the wet shear strength of the resultant plywood by 105.4%;
(2) The water resistance and the crosslinking density of the adhesive were improved because of the following mechanisms: (a) a crosslinking structure formed through the reaction between the TR and the soy protein molecules; (b) An interpenetrated network formed through the crosslinking protein molecules and the self-crosslinking TR molecules; (c) NaOH in TR made the soy protein more soluble and more ionic amino acids, which increased the reactive sites of soy protein with crosslinkers, thus increasing the crosslinking density;
(3) The viscosity improved, which was beneficial for the adhesive distribution during the hot press process. This formed a stronger interlock with the wood surface, which resulted in a better water resistance and bonding strength obtained in the resultant adhesive; and
(4) The addition of 10 wt % TR in SPA decreased the bonding strength of the resultant adhesive because the quantity of unfolded protein molecular chains that caused the resultant adhesive to have a very high viscosity. This caused the adhesive to adhere the wood surface non-uniformly, which did not give the resultant plywood effective bonding strength.

## Figures and Tables

**Table 1:** The physical properties of TR.

Resin	Solid Content	Viscosity	pH
TR	57.1 ± 0.3%	970 ± 9 mPa·s	10.0 ± 0.2

**Table 2:** The viscosity and pH value of different adhesives.

Adhesive Samples	SPA	SPA-TR2.5	SPA-TR5	SPA-TR7.5	SPA-TR10
Viscosity (mPa·s)	111,900 ± 1357	45,800 ± 689	71,010 ± 476	94,790 ± 413	105,512 ± 978
pH value	7.2 ± 0.2	7.5 ± 0.1	7.9 ± 0.2	8.2 ± 0.1	8.6 ± 0.1

**Table 3:** The shear strength and residual rate of the different adhesive samples.

Adhesive Samples	SPA	SPA-TR2.5	SPA-TR5	SPA-TR7.5	SPA-TR10
Dry shear strength (MPa)	1.24 ± 0.10	1.83 ± 0.15	2.27 ± 0.13	2.02 ± 0.11	1.74 ± 0.12
Wood failure	10%	40%	70%	50%	30%
Wet shear strength (MPa)	0.55 ± 0.02	0.87 ± 0.03	1.13 ± 0.08	0.98 ± 0.04	0.79 ± 0.05
Wood failure	0	30%	50%	30%	10%
Residual rate	61.8 ± 0.6%	81.2 ± 1.0%	83.7 ± 0.4%	79.5 ± 0.4%	72.3 ± 0.4%
